# Cost-effectiveness analysis of the treatment with compressive therapy in the healing of venous ulcers *

**DOI:** 10.1590/1518-8345.6017-3840

**Published:** 2023-03-27

**Authors:** Sarah Lopes Silva Sodré, Glycia de Almeida Nogueira, Alcione Matos de Abreu, Cristiano Bertolossi Marta, Antônio Augusto de Freitas Peregrino, Roberto Carlos Lyra da Silva

**Affiliations:** 1 Hospital Universitário Graffre Guinle, Centro de Terapia Intensiva, Rio de Janeiro, RJ, Brasil; 2 Hospital Central da Aeronáutica, Rio de Janeiro, RJ, Brasil; 3 Universidade Federal do Estado do Rio de Janeiro, Rio de Janeiro, RJ, Brasil; 4 Universidade Federal do Estado do Rio de Janeiro, Departamento de Enfermagem Médico-Cirúrgica, Rio de Janeiro, RJ, Brasil; 5 Universidade do Estado do Rio de Janeiro, Departamento de Enfermagem Fundamental, Rio de Janeiro, RJ, Brasil; 6 Universidade Veiga de Almeida, Faculdade de Enfermagem, Rio de Janeiro, RJ, Brasil; 7 Universidade do Estado do Rio de Janeiro, Departamento de Ciências Radiológicas, Rio de Janeiro, RJ, Brasil

**Keywords:** Varicose Ulcer, Leg Ulcer, Compression Bandages, Bandages, Wound Healing, Technology Assessment, Biomedical, Úlcera Varicosa, Úlcera de la Pierna, Vendajes de Compresión, Vendajes, Cicatrización, Evaluación de la Tecnología Biomédica, Úlcera Varicosa, Úlcera da Perna, Bandagens Compressivas, Bandagens, Cicatrização da Tecnologia Biomédica

## Abstract

**Objective:**

to analyze the cost-effectiveness and calculate the incremental cost-effectiveness ratio of multilayer compressive treatment in relation to inelastic (Unna boot and short stretch) therapy according to the current literature.

**Method:**

quantitative study about cost-effectiveness through modeling with the aid of TreeAge® software for construction of the decision tree. The anticipated assumptions were obtained by using secondary literature data to estimate the cost and effectiveness of the assumed parameters. A systematic literature review with meta-analysis was performed for this end.

**Results:**

the decision tree after Roll Back showed that the multilayer therapy dominated the alternatives in the base case, representing an intermediate cost per application, although with the highest effectiveness. The cost-effectiveness analysis graph also showed extended dominance of the Unna boot in relation to the short stretch bandage. The sensitivity analysis showed that multilayer bandage remains a more cost-effective alternative, within the threshold of willingness to pay.

**Conclusion:**

the most cost-effective alternative was multilayer bandage, considered the gold standard in the literature. The second most cost-effective alternative was the Unna boot, the most used therapy in Brazil.

## Abstract (es)

**Objetivo:**

analizar el costo-efectividad y calcular la relación costoefectividad incremental del tratamiento multicapa compresivo con respecto al inelástico (bota de Unna y estiramiento corto) según la literatura actual.

**Método:**

estudio cuantitativo de costo-efectividad a través de un modelo con ayuda del software TreeAge® para la elaboración del árbol de decisión. Los supuestos anunciados se obtuvieron mediante el uso de datos secundarios de la literatura para estimar el costo y la efectividad de los parámetros asumidos. Para ello, se realizó una revisión sistemática de la literatura con metaanálisis.

**Resultados:**

el árbol de decisión, después del Roll Back, mostró que la terapia multicapa prevaleció sobre las alternativas en el caso base, presentó un costo intermedio por aplicación, pero obtuvo la mayor efectividad. El gráfico del análisis de costo-efectividad también demostró que había un dominio extendido de la bota de Unna sobre el vendaje de estiramiento corto. El análisis de sensibilidad reveló que el vendaje multicapa sigue siendo la alternativa con mayor costoefectividad, dentro del umbral de disposición a pagar.

**Conclusión:**

la alternativa con mayor costo-efectividad fue el vendaje multicapa, considerado estándar de oro en la literatura. La segunda alternativa con mayor costo-efectividad fue la bota de Unna, la terapia más utilizada en Brasil.

## Abstract (pt)

**Objetivo:**

analisar a custo-efetividade e calcular a razão de custoefetividade incremental do tratamento compressivo multicamadas em relação ao inelástico (bota de Unna e curto estiramento) de acordo com a literatura atual.

**Método:**

estudo quantitativo sobre custo-efetividade por meio de modelagem com auxílio do software TreeAge® para a construção da árvore de decisão. Os pressupostos anunciados foram obtidos pelo uso de dados secundários de literatura para estimativa do custo e efetividade dos parâmetros assumidos. Para tal, foi realizada uma revisão sistemática de literatura com metanálise.

**Resultados:**

a árvore de decisão, após Roll Back mostrou que a terapia multicamadas dominou as alternativas no caso-base, representando custo intermediário por aplicação, porém, com a maior efetividade. O gráfico da análise de custo-efetividade também mostrou uma dominância estendida da bota de Unna em relação à bandagem de curto estiramento. A análise de sensibilidade mostrou que a bandagem multicamadas permanece como alternativa mais custo-efetiva, dentro do limiar de disposição para pagar.

**Conclusão:**

a alternativa com maior custo-efetividade foi a bandagem multicamadas, considerada padrão ouro na literatura. A segunda alternativa mais custo-efetiva foi a bota de Unna, terapia mais utilizada no Brasil.

**Highlights**

(1) One of the few cost-effectiveness studies of compressive therapies in Brazil.

(2) It showed that the multilayer system is the most cost-effective among those evaluated.

(3) It also showed that the Unna boot is the second most cost-effective method.

(4) innovative study method for nurses.

## Introduction

Venous Ulcer (VU) is the result of an advanced stage of chronic venous disease and is caused by venous hypertension complications. This type of injury is characterized by chronicity and can generate physical dysfunction, pain and problems for performing daily activities. The recurrence rate is uncertain and can vary from 26% to 70%, which shows the need for interdisciplinary monitoring and care until the end of life, therefore with the possibility of resulting in increased health costs ^(1- 2)^ . 

Data show that approximately 75% of the leg ulcers are of venous etiology, affect nearly 1% of the world population, and are more prevalent in older adults ^(1, 3)^ . In Brazil, this rate affects between 3% and 4% of the population, with higher predominance among women over 65 years old ^(1, 3- 4)^ . 

There is already international consensus that compressive therapy is the gold standard for the treatment of venous ulcers, being contraindicated in patients with severe peripheral arterial disease. Compression makes it possible to reverse venous hypertension at the level of the superficial veins in the lower limbs and, according to experts, ideal therapeutic levels should fluctuate between 35 and 45 mmHg in the ankle region and the patient should be encouraged to walk ^(1, 5- 7)^ . 

Among the compression methods available and recommended for use in venous ulcers are inelastic and elastic compression therapies. As an example of inelastic therapy we can mention short stretch bandages and the Unna paste bandage (Unna boot), the method most used in Brazil and in some other countries such as the United States of America (USA). Among the elastic compression methods, it is worth mentioning the multilayer bandage system, which can be found with two, three or four layers, producing a cumulative effect and providing constant compression ^(8)^ . 

A Cochrane review carried out in 2012 concluded that the most effective compressive therapy method in the treatment of venous ulcers is the multilayer system when compared to other therapies such as Unna boot and short stretch bandage; however, this system has a higher unit cost than other technologies, requiring studies to evaluate the cost-effectiveness ratio ^(7, 9)^ . 

A number of studies show that the USA spends approximately US$ 25 billion per year on the treatment of chronic injuries of vascular etiology ^(10)^ . In Brazil, these lesions are responsible for high morbidity and mortality rates, exerting a significant impact on the costs, which are still little explored in scientific studies ^(1, 8)^ . 

Precisely because of their chronicity, high prevalence and need for long-term care, they are considered a worldwide public health problem. These injuries impair quality of life and productivity in the people affected by them and generate a socioeconomic impact, which needs to be considered in countries with universal health systems such as Brazil, despite the fact that they are not associated with high mortality rates ^(6, 11- 12)^ . 

In the search for efficiency in the allocation of financial resources, for at least 20 years, countries with universal health systems such as the United Kingdom have sought to inform their decisions and choices based on the best scientific evidence available. Although still incipient in Brazil, Health Technology Assessments (HTAs) have been increasingly used, not only among managers, but also among health professionals, as a way to reduce uncertainties around the best available alternatives, especially in cases involving countless options and high-cost technologies.

In this sense, HTA studies have become increasingly common and indispensable among decision-makers, such as cost-effectiveness studies that represent a widely used method, as they allow for comparisons between different alternatives considering their costs and effectiveness (consequences), as not always a more effective option justifies the cost of its use for the payer ^(13)^ . 

In Brazil, it is necessary to discuss why the Unna boot is the type of compressive therapy most used to treat patients with ulcers, to the detriment of others such as multilayer and short stretching bandages, much more used in other countries. Are such choices being based only on the cost of the alternatives and personal preferences or is effectiveness also considered at the same time?

Thus, the structured research question is as follows: Is multilayer compression therapy more cost-effective in the healing of venous ulcers than the Unna boot and short-stretch bandage inelastic compression therapies? This study aims at analyzing the cost-effectiveness and calculate the incremental cost-effectiveness ratio (ICER) of the multilayer compressive treatment in relation to the inelastic (Unna boot and short stretch) therapy

according to the current literature.

## Method

### Type of study

This is a quantitative study about cost-effectiveness a cost-effectiveness study based on modeling, using the TreeAge® software, 2021. The decision model used in this study was the decision tree with use of data from a systematic review with meta-analysis.

### Scenario

The hypothetical scenario was the Wounds medical office, a place for the application of compressive therapy in health services.

### Study period

The secondary data collection procedure took place between 2019 and 2021. The statistical and cost- effectiveness analyses were performed in 2021.

### Population and criteria to select and define the sample

The model was populated by a hypothetical cohort of adult patients, of both genders, with venous leg ulcers with indication for compressive therapy, who underwent treatment with any of the compression methods analyzed (multilayer, Unna boot, short-stretch). The sample was obtained based on data from studies included in the systematic review and in the data meta-analysis.

### Data collection and instruments used

The technologies evaluated were multilayer bandage, Unna boot and short-stretch bandage, all characterized as compressive therapy methods, used in the treatment of venous ulcers.

The secondary data used to estimate effectiveness in elaboration of the model were obtained from a systematic review of the “rapid review” type, part of the main author’s PhD thesis ^(14)^ . It is worth mentioning that the search was carried out in May 2019 and revised in 2021, in the Medline database via *PubMed,* as well as in the Virtual Health Library ( *Biblioteca Virtual de Saúde*, BVS) portal and in the Cochrane Library, that the selection flow to select the papers followed the PRISMA (Preferred Reporting Items for Systematic reviews and Meta-Analyses) guidelines, that the papers retrieved were exported to the Rayyan online software for selection of the studies, counting on the use of the risk of bias assessment tool (Cochrane Risk of Bias Tool), through the Cochrane systematic review software, Review Manager (RevMan) version 5.4.1. ( https://revman.cochrane.org/). The research question was structured using the PICO acronym, as follows: Population (P): People with chronic venous ulcers; Intervention (I): Multilayer elastic compression therapy; Control (C): Unna boot and short- stretch bandage Inelastic compression therapies, and Outcomes (O): Healing and lower cost. After cataloging 

the terms and analyzing each PICO arm, the search strategy was prepared in each of the databases used.

In relation to the design, all 12 studies included in the meta-analysis comprised randomized clinical trials. In all these studies, it was not possible to shield the participants and professionals, considering the visual characteristics of each compressive therapy ^(15- 26)^ . 

**Figure 1:**
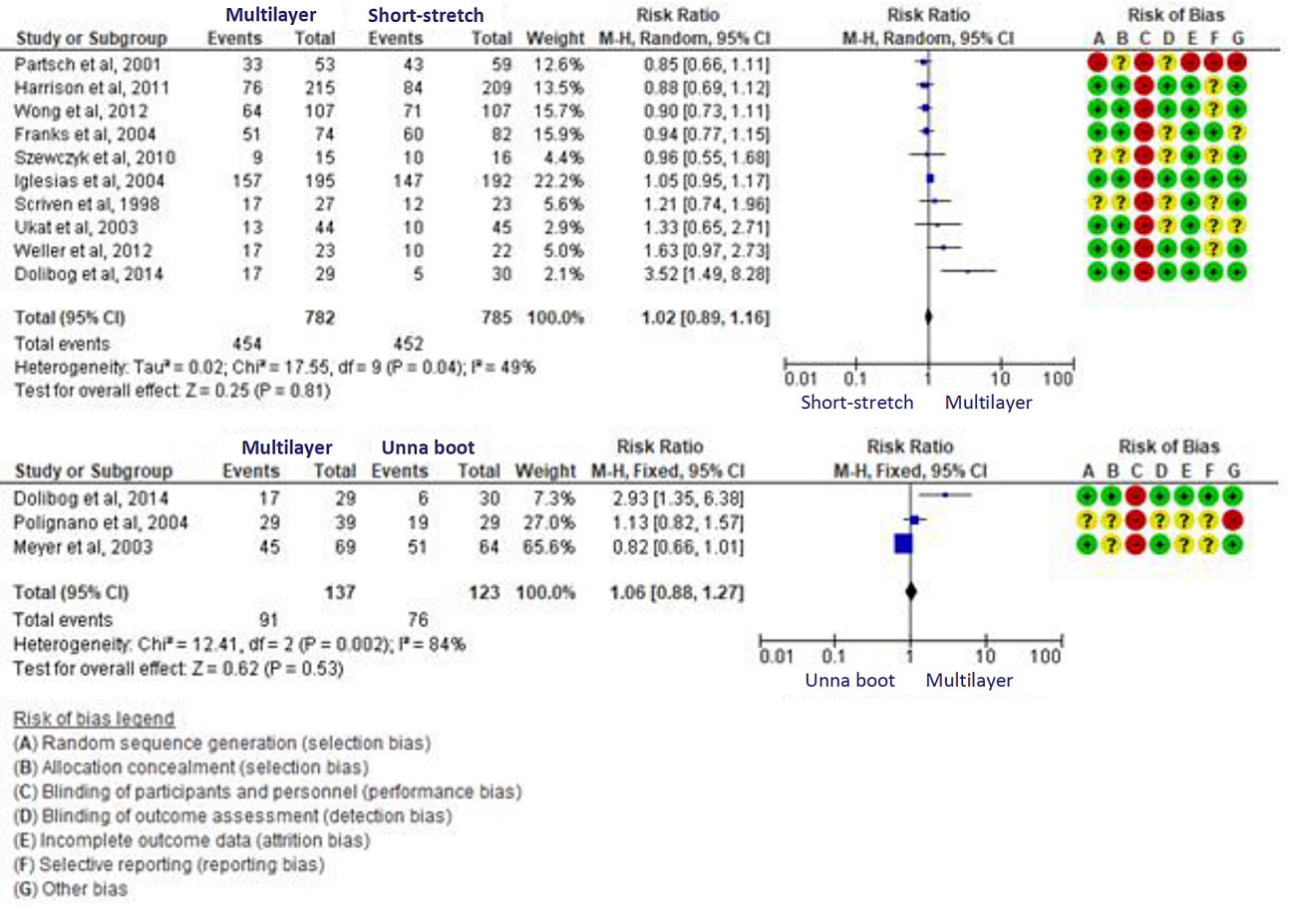
Forest Plot of the multilayer versus short-stretch and multilayer versus Unna boot meta-analysis for the healing outcome

Note: Forest Plot study developed with the aid of the RevMan 5.4 software from Cochrane

The primary outcome analyzed was ulcer healing and the secondary outcomes were healing time and reduction percentage. Some of the studies evaluated presented the data in different periods, and the mean was calculated. For the primary outcome, the sample number obtained in the multilayer comparison *versus* Unna boot was 264 ulcers and for the multilayer comparison *versus* short-stretch it was 1,567, as shown in Figure 1. 

Most of the studies evaluated (n=10) compared multilayer bandage with short-stretch. Only 3 studies compared multilayer with Unna boot. Of these, 1 compared 5 compressive therapy methods. Therefore, the data were used both for the intervention (multilayer) and for the two comparators (short-stretch and Unna boot) ^(15- 26)^ . 

The meta-analysis of these studies revealed that there is no significant difference in effectiveness in the treatment of venous ulcers when comparing multilayer bandage *versus* short-stretch (RR 1.02; 95% CI [0.89, 1.16]), as well as in the multilayer *versus* Unna boot comparison (RR 1.06; 95% CI [0.88, 1.27]). 

With regard to the multilayer *versus* Unna boot comparison, the first increases the likelihood of healing by 6%, in the worst case scenario, it may reduce it by 12% (RRR=12%) and in the best case, increase by 27% (ERR=27%). In the multilayer *versus* short-stretch comparison, the first shows effectiveness when compared to the second, increasing by 2% the possibility of healing, in the worst case scenario reducing it by 11% (RRR=11%) and, in the best estimate, increasing it by 16% (ERR=16%). 

For calculation of the secondary outcomes, in the case of continuous and non-dichotomous variables, the result was obtained by the difference between the means and not through the relative risk. The “healing time” secondary outcome showed that in the multilayer *versus* short-stretch comparison, the first reduces this time by mean of 7.34 days, increasing this time in the worst case scenario by 7.46 days and, in the best case scenario, reducing it by 22.14 days. However, in the comparison with the Unna boot, this outcome was negative for multilayer, as it increased the healing time by a mean of 11.55 days (in the worst case scenario increasing it by 49.25 and, in the best case, reducing by 26.16 days). 

The mean healing times allow calculating the total cost with the treatment of each therapy, by dividing the mean healing time by the maximum frequency of dressing exchange (7 days) and multiplying by the costs of each dressing.

Calculation of the mean healing time with the multilayer bandage was obtained with the inclusion of all 6 studies that evaluated healing time with the use of this therapy, both in comparison with Unna boot and the short-stretch bandages, being equal to 77.05 days. For the Unna boot option, only two studies evaluated the healing time, obtaining a mean of 77 days. This time was longer for short-stretch: 83.65 days.

Identification of the cost items related to the materials used for use of the technologies was carried out based on an observational study conducted in 2006, in which a survey of the cost of the procedure with the Unna boot was carried out; the method most used in Brazil, with an adaptation of the cost items used in the use of other technologies ^(27)^ . 

Consultation of the evaluated technologies available in Brazil and with current records was carried out on the ANVISA website, and the monetary values of the cost items were obtained through the search in the *Brasíndice* pharmaceutical guide, volume 56, No. 963, from December 18 ^th^, 2020 and in the VideoFarma – SIMPRO electronic table, serial number: 11183 and imputed to Microsoft Excel spreadsheets. The costing technique was macro-costing, which is why the costs related to the professional workforce were not considered. Another aspect taken into consideration was the similarity between the technologies with regard to the exchange frequency and time spent on the procedure and the fact that macro-costing was used. 

The meta-analysis performed to estimate the likelihood of healing for the short-stretch bandage was compared to multilayers, and the likelihood of healing for the short-stretch bandage was 0.5758. The likelihood of healing for the multilayer bandage was compared to the Unna boot, which presented a value of 0.6169, with a sum of 0.06 referring to the chance for healing presented by the multilayer bandage in this comparison (0.6169 + 0.06 – RR 1.06) being established at 0.6769. For all technologies, it was assumed in the model that the probability of healing can vary between 0% and 100%. All the analyses were performed with the aid of the RevMan 5.4 software.

The weekly cost in dollars for the use of multilayer bandage was US$ 79.86. For Unna boot use this value was lower, US$ 39.39 and it was the highest for short-stretch bandage, US$ 65.92 (with inclusion of the bandage and protective foam) and US$ 20.24 (without bandage or foam). Both bandage and foam can be washed; however, we will not go into the merits of the amount of bandages and washes required, we have adopted US$ 93.08 as the mean cost.

It is important to highlight that the study was carried out in the pre-pandemic period, in which the costs for acquiring technologies, inputs and human resources were not as pressured as in the pandemic period, which directly affected the supply chain and the economic and industrial health complex, which caused these costs to increase considerably in relation to the pre-pandemic period.

### Data treatment and analysis

**Figure 2:**
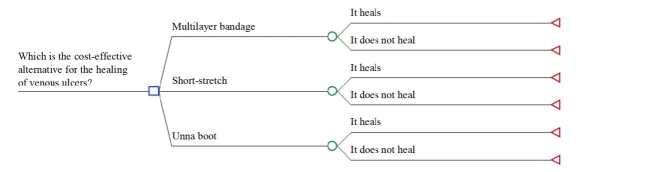
Structure of the decision tree model using the TreeAge® software

In the model proposed in Figure 2, we consider healing as the critical outcome of interest in this analysis. The possibility of other model structures to be tested, such as options to the model proposed, to address possible structural uncertainties will not be considered in the sensitivity analysis. 

Parametric uncertainties were treated by probabilistic sensitivity analysis, from performance of second- order Monte Carlo simulations (10,000 simulations), considering the cost variables, for which the Gamma distributions were assigned, and the probability and effectiveness variables, assigning Beta distributions. The α (alpha) and β (beta) of the Beta distributions and the α and λ (lambda) of the Gamma distributions were estimated from the means and standard deviations of the variables used in the analyses.

It should be noted that reporting of the results of the economic analysis followed the recommendations set forth in the updated version of CHEERS ( *Consolidated Health Economic Evaluation Reporting Standards*). The new CHEERS declaration, published in 2022, replaces the previous guidance and has a 28-item checklist ^(28)^ . 

### Ethical aspects

The study in question does not directly involve human beings because it is carried out through secondary sources, being submitted to and approved by *Plataforma Brasil* with waiver of an Ethics and Research Committee through Certificate of Presentation of Ethical Appreciation (CAAE) 16947419.0.00 00.5285. 

### Study protocol

The following assumptions were presumed in the model:

1- The analysis perspective was the Health Operators’.
2- The time horizon was 1 year.
3- No discount or inflation rate was applied for costs and effectiveness in view of the analysis short time horizon, following the recommendations of the Methodological Guidelines for carrying out economic analyses in health ^(13)^ .
4- Effectiveness was measured by the probability of healing occurring.
5- The bandage exchange frequency was 7 days.
6- Only the direct costs with the necessary inputs for the application of dressings and technologies were considered.
7- The mean cost for application of the multilayer bandage was US$ 79.86; for Unna boot it was US$ 39.39, and for short-stretch it was US$ 93.08.
8- The mean healing time using multilayer bandage was 77.05 days, for Unna boot it was 77, and for short-stretch it was 83.75 days.
9- The probability of healing was the effectiveness measure for all the technologies analyzed.
10- The effectiveness assumed for multilayer bandage was 0.6779, for Unna boot it was 0.6179, and for short-stretch it was 0.5758, varying from 0.001% to 100%.
11- Willingness to pay (WTP) was estimated at 01 GDP (Gross Domestic Product) per capita considering the year 2018, estimated at US$ 6,186.71 according to the Brazilian Institute of Geography and Statistics ( *Instituto Brasileiro de Geografia e Estatística*, IBGE)^*^.
12- The amounts were converted to dollars using the commercial dollar quotation for purchase from the Central Bank: US$ 5.43^**^.

## Results

The results of the model after *Roll Back* show that the compressive therapy using multilayer bandages dominated the other two alternatives in the base case, representing in the model the intermediate cost alternative between the highest (short-stretch) and lowest (Unna boot) cost *per* application (US$ 53.96), although with the highest effectiveness among the alternatives analyzed (46%). 

**Figure 3:**
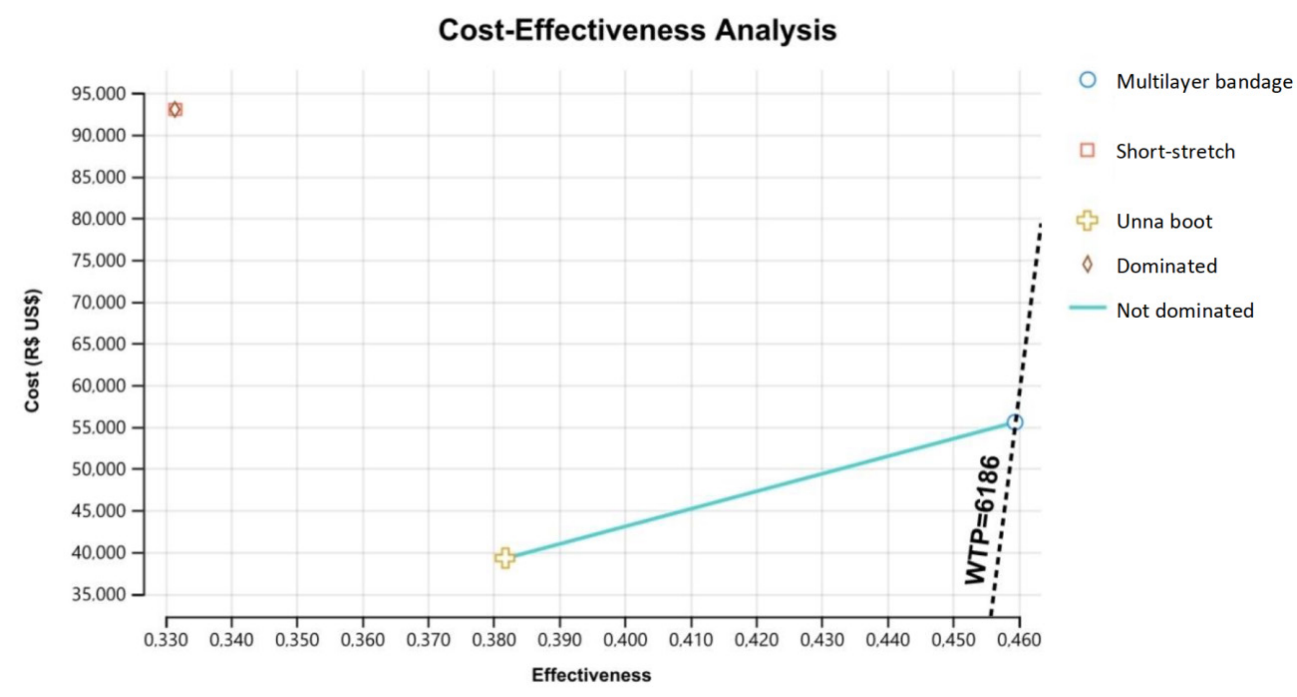
Cost-Effectiveness analysis graph

Source: Cost-Effectiveness Model after the Decision Tree proposed by the author, using the TreeAge® software

The cost-effectiveness analysis graph (Figure 3) shows that there was a weak (or extended) dominance of the Unna boot in relation to the short-stretch bandage. It should be noted that the line that unites both technologies that dominated the short-stretch bandage, in this case, the multilayer bandage and the Unna boot (extended dominance), shows that either of the two technologies can be cost-effective depending on the willingness to pay threshold. 

The cost-effectiveness analysis presented an incremental cost-effectiveness ratio (ICER) of US$ 185.43 in favor of multilayer bandage, within the willingness to pay threshold defined in the base case at US$ 6,186.71.

Probabilistic sensitivity analysis was performed from 10,000 Monte Carlo simulations, also with the aid of the TreeAge ®software. For the cost variables, Gamma distributions were assigned and for probabilities and effectiveness, Beta distribution, following the recommendations of the Methodological Guidelines for carrying out economic evaluations in health ^(13)^ . 

In the proposed economic model designed to evaluate effectiveness of the treatment of venous ulcers, many parameters, which represent, for example, the injury healing probability, can assume different values, reflecting a mean among the various spectra of severity of chronic venous failure (CVF), that is, the parameter has variation in the target population and that more than two alternatives were compared in the base case.

The incremental cost-effectiveness dispersion graphs and the cost-effectiveness acceptability curve are presented below, considered as the most important in cost-effectiveness analysis and, therefore, the most used for interpretation of probabilistic analyses. Incremental cost-effectiveness graphs were plotted comparing Unna boot (extended dominance) and multilayer bandage at baseline, which in the analysis proved to be the most cost-effective alternative.

**Figure 4 -:**
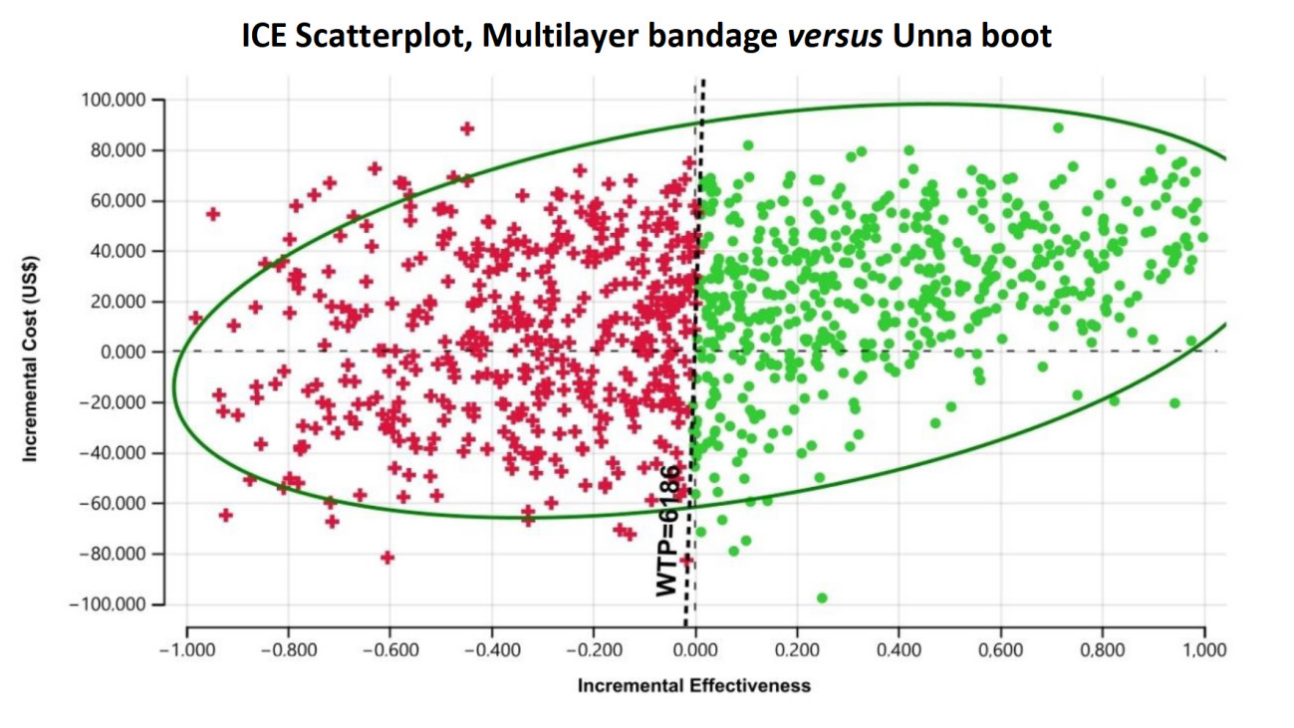
Incremental cost-effectiveness graph of multilayer technologies *versus* Unna boot with the use of the TreeAge® software

In the incremental cost-effectiveness dispersion chart, shown in Figure 4, each colored point represents each of the 10,000 iterations, formed by the incremental cost (y-axis) and the incremental effectiveness (x-axis). This graph was plotted using as a comparator the intermediate cost alternative in the base case (Unna boot) and at baseline, the dominant alternative, multilayer bandage. 

Through this graph it is possible to identify the proportion of iterations that agree with the mean/deterministic value. A result with significant uncertainty can be noticed since, depending on the iteration, the result can be dominant, dominated or a conflictive choice and everything will depend on willingness to pay, as already seen in the cost-effectiveness graph (Figure 3), in which it is possible to observe that both alternatives (multilayer bandage and Unna boot) are joined by an oblique line which informs us that any of the technologies can be cost-effective. 

Also with respect to the incremental cost-effectiveness graph (Figure 4), the darker dotted line that crosses the upper right and lower left quadrants, I and III respectively, represents the willingness to pay threshold. Therefore, all iterations that are below this line should be considered as cost-effective. It is important to note that this dotted line divides quadrants I and III, forming another two components, thus making a total of 6 components. 

It is to be noted that this graph is plotted on a Cartesian plane, in which the quadrants (I, II, III and IV) are counted from right to left, forming two upper and two lower quadrants. Quadrant I refers to the iterations, in which costs and effectiveness are high, with the possibility of being cost-effective depending on willingness to pay.

In quadrant II, the iterations represent an increase in cost that is not accompanied by the same proportion of increased effectiveness. In quadrant III, cost and effectiveness are lower, and the cost-effective technology may also depend on willingness to pay. Finally, in quadrant IV, the iterations represent the lowest costs and highest effectiveness. The ellipse represents 95% confidence.

It is therefore easy to understand that the iterations in quadrants I and III are those that will represent the “Trade Off”, in which the technology can be cost-effective depending on how much a person is willing to pay, so that such a provision is higher than the ICER.

Quadrants II and IV leave no doubt: the iterations in quadrant II speak in favor of rejecting the technology, while when located in quadrant IV, one should speak of incorporating the technology. However, the decision will depend on the proportion of iterations that are located in the quadrants considered as possible to be cost- effective, in this case I, III and IV, while considering willingness to pay, of course.

In the comparison between multilayer and Unna boot, we can clearly see a more homogeneous distribution of the iterations between quadrants and components of the Cartesian plane that helps reinforce the results of the model after *Roll Back* and the cost-effectiveness analysis, which showed the superiority of multilayer bandage and the extended dominance of Unna boot over short-stretch bandages. 

The frequency of iterations that place the multilayer bandage as a cost-effective option in relation to the Unna boot, depending on willingness to pay, totals 68.65%. The proportion of iterations favorable to multilayer bandage, as it represents an increase in effectiveness and lower cost, was 9.26%. Only 22.4% of the iterations were unfavorable to multilayers.

In relation to the comparison between multilayer and short-stretch bandage, the proportion of favorable iterations is 55.57%. Only 14.82% of the iterations are unfavorable to the multilayer bandage, once again ratifying the extended dominance of the Unna boot over the short-stretch bandage in terms of cost-effectiveness.

**Figure 5:**
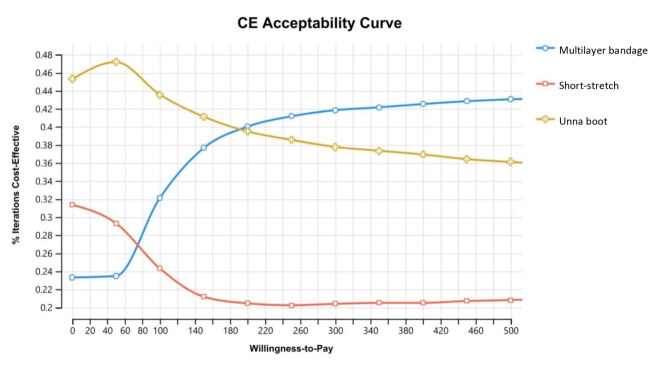
Cost-Effectiveness Acceptability Curve graph created using the TreeAge® software

Figure 5 shows the acceptability curve corresponding to the cost-effectiveness of the alternatives analyzed in the base case. In it, it is possible to identify that, for “willingness to pay” equal to or less than US$ 190.00, the Unna boot is more likely to be more cost-effective than the other alternatives (38%). 

From approximately US$ 200.00, multilayer bandage surpasses Unna boot and assumes the position of the alternative with a higher probability of being cost-effective in the base case. The intersection point between the Unna boot curves and the multilayer bandage represents the *Q* point. At this point, both alternatives are equally likely to be cost-effective according to the willingness to pay threshold. 

From point *Q*, as willingness to pay increases, multilayer bandage increases its probability of being the most cost-effective alternative, reaching a maximum probability of approximately 46%. It is important to note that, from approximately US$ 250.00, there is no point in increasing willingness to pay, as there will be no increase in the likelihood of this alternative being even more cost-effective. 

In scenarios where willingness to pay is less than US$ 70.00, short-stretch bandages were only able to overcome multilayer bandages in terms of their probability of being cost-effective, although outdone by the Unna boot in these scenarios, in the case of the alternative with a higher probability of being cost-effective when compared to the others.

## Discussion

In this paper, the multilayer bandage was the alternative with the best cost-effectiveness (ICER = US$ 185.44) to the detriment of other technologies, as well as in the study developed by English researchers in 2004, where they only used one comparator: short-stretch bandage. In this study, as two comparators were used, it was possible to evaluate the second most cost-effective method. Thus, the Unna boot (ICER = US $0) presented extended dominance in relation to the short-stretch bandage (ICER = US$ 318.82) ^(20)^ . 

The effectiveness-related data in this study were based on 9 studies for the multilayer *versus* short-stretch comparison. Due to the scarcity of studies evaluating effectiveness of the Unna boot, the meta-analysis that compared multilayer bandage with Unna boot was only performed with 3 studies. 

A Cochrane review conducted in 2012 evaluated various types of compressive therapy. In the multilayer *versus* short-stretch comparison for the healing outcome, five studies were included in the meta-analysis, totaling 797 ulcers and obtaining a RR of 0.96 [0.88-1.05]. Thus, the multilayer bandage was considered as the most effective alternative. In the comparison with Unna boot there was no significant difference between these therapies ^(9)^ . 

Another meta-analysis that compared multilayer with short-stretch bandages was carried out by Brazilian researchers in 2018, with inclusion of 7 articles and 1,446 participants, resulting in a RR of 1.11 [0.99-1.24], which shows the need for more clinical trials that provide consistent effectiveness data, as well as for analyses that compare the costs to the effectiveness of such technologies ^(29)^ . 

Important factors to be considered in the comparison between compression systems are size of the ulcer and its duration, which can slow down the healing process. In Brazil, a clinical trial that compared Unna boot to simple elastic bandages showed that the former provided better results in lesions larger than 10 cm ^2^ while the elastic bandages showed better results in smaller ulcers. This evidences the need for clinical trials in patients with large VUs, something very common in our country, when compared to the injuries of individuals living in First-World countries ^(8- 9)^ . 

All models are subjected to uncertainties related to the anticipated assumptions. In order to minimize the possible errors arising from such uncertainties, imputed in the decision tree model, sensitivity analyses were performed in accordance with the recommendations of the Brazilian Economic Assessment Guidelines.

The probabilistic sensitivity analysis showed that, considering the willingness to pay adopted in this study, as 1 GDP *per capita*, the iterations favorable to multilayer bandage exceeded the other technologies, in a higher proportion when compared to the short-stretch bandage and in a smaller proportion in relation to the Unna boot, which can be justified by the extended effectiveness of this technology, which helps to reinforce and reduce the uncertainties of the parameters imputed in the model. 

In relation to the acceptability curve, we can observe that the Unna boot exceeds the other technologies in a scenario where willingness to pay is lower and, as this value increases, effectiveness of the multilayer bandage is also increased, showing an effectiveness level similar to the Unna boot at point *Q* (approximately US$ 190.00). The difficulty locating robust studies that would allow estimating the effectiveness of the technologies evaluated with precision is emphasized, mainly tests developed in our country, which would allow evaluating bandages in the real scenario of Brazilians with VU. 

It was not possible to carry out a budget impact analysis that could help further reduce the uncertainties regarding feasibility and sustainability of the possible incorporation of multilayer bandages.

Considering the lack of information in the literature to support the economic analysis, the effectiveness data were considered from the likelihood of healing and the costs for SUS-related unofficial sources, transferring the perspective to the private sector (health operators) and imposing structural limitations for not considering other possible health conditions and eventual transitions, which might have been better explored in a microsimulation. It is important to highlight that the effectiveness was obtained from international studies due to the nonexistence of RCTs using these comparators in the Brazilian population, which can differ when transferred to our reality.

## Conclusion

This research allows extrapolating the nurses’ views beyond patient care and combining care based on the best evidence with the managerial capacity, already common and intrinsic to these professionals, projecting another perspective of studies that can be carried out by this category, allowing for the expansion of horizons for those who are interested in undertaking the field of HTA studies.

This economic analysis concluded that multilayer bandage is the most cost-effective alternative to treat chronic wounds of a venous etiology affecting the lower limbs, with an ICER of US$ 185.44, dominating the alternatives compared in the base case.

The extended dominance of the Unna boot in relation to the short-stretch bandage should be considered in the analysis, taking into account that this technology is the most used for VU treatment in the Unified Health System ( *Sistema Único de Saúde*, SUS), probably because of its low cost, thus enabling greater supply and coverage in terms of treatment. 

It was possible to assert that, for the treatment of venous ulcers, both multilayer bandages and Unna boots can be cost-effective, depending on the managers’ willingness to pay. Multilayer bandages remain the most cost-effective alternative, in line with the results reported in the world literature.

After the probabilistic sensitivity analysis, multilayer bandages remained as a more appealing alternative from the cost-effectiveness point of view, within the willingness to pay threshold of 1 GDP *per capita*, stipulated at US$ 6,186.71, taking 2018 as reference. It is worth noting that the option for 1 GDP *per capita* is in line with what is suggested by the Methodological Guidelines for carrying out economic evaluations in health. 

It is important to ratify that, regardless of the technology to be used, compressive therapy remains the most effective method for VU treatment, provided that it is properly indicated and implemented, with nurses as the professionals who deserve to be highlighted in the multidisciplinary team for the treatment of these patients.

## Footnotes

*
https://sidra.ibge.gov.br/tabela/6784#/n1/all/v/9812/p/all/d/v9812%202/l/v,,t+p/resultado

**
US Dollar quotation = R$ 5.4295, on 01/22/2021
